# Supplementary material for: Computer assisted detection of axonal bouton structural plasticity in in vivo time-lapse images
Source: eLife. 2017 Oct 23;6:e29315. doi: 10.7554/eLife.29315 (PMC5675596; doi:10.7554/eLife.29315)
Supplement: Transparent reporting form [file elife-29315-transrepform.docx]

***eLife’s* transparent reporting form**

We encourage authors to provide detailed information *within their submission* to facilitate the interpretation and replication of experiments. Authors can upload supporting documentation to indicate the use of appropriate reporting guidelines for health-related research (see [EQUATOR Network](http://www.equator-network.org/%20)), life science research (see the [BioSharing Information Resource](https://biosharing.org/)), or the [ARRIVE guidelines](http://www.plosbiology.org/article/info:doi/10.1371/journal.pbio.1000412) for reporting work involving animal research. Where applicable, authors should refer to any relevant reporting standards documents in this form.

If you have any questions, please consult our Journal Policies and/or contact us: [editorial@elifesciences.org](mailto:editorial@elifesciences.org).

**Sample-size estimation**

- You should state whether an appropriate sample size was computed when the study was being designed
- You should state the statistical method of sample size computation and any required assumptions
- If no explicit power analysis was used, you should describe how you decided what sample (replicate) size (number) to use

Please outline where this information can be found within the submission (e.g., sections or figure legends), or explain why this information doesn’t apply to your submission:

The goal of this Tools and Resources paper is to describe and validate bouton detection methodology implemented in BoutonAnalyzer software. Therefore, no explicit power analysis was performed. Sample sizes used for validation are listed in Results, Materials and Methods, legends to Figures 2 and 5, and Table 1.

**Replicates**

- You should report how often each experiment was performed
- You should include a definition of biological versus technical replication
- The data obtained should be provided and sufficient information should be provided to indicate the number of independent biological and/or technical replicates
- If you encountered any outliers, you should describe how these were handled
- Criteria for exclusion/inclusion of data should be clearly stated
- High-throughput sequence data should be uploaded before submission, with a private link for reviewers provided (these are available from both GEO and ArrayExpress)

Please outline where this information can be found within the submission (e.g., sections or figure legends), or explain why this information doesn’t apply to your submission:

Only technical replicates are relevant for this study. Details are provided in Figure 2 – figure supplements 1 and 2, Quantifying precision of bouton detection methodology subsection of Materials and Methods, and Effects of imaging conditions on bouton analysis subsection of Results.

**Statistical reporting**

- Statistical analysis methods should be described and justified
- Raw data should be presented in figures whenever informative to do so (typically when N per group is less than 10)
- For each experiment, you should identify the statistical tests used, exact values of N, definitions of center, methods of multiple test correction, and dispersion and precision measures (e.g., mean, median, SD, SEM, confidence intervals; and, for the major substantive results, a measure of effect size (e.g., Pearson's r, Cohen's d)
- Report exact p-values wherever possible alongside the summary statistics and 95% confidence intervals. These should be reported for all key questions and not only when the p-value is less than 0.05.

Please outline where this information can be found within the submission (e.g., sections or figure legends), or explain why this information doesn’t apply to your submission:

- R2 values are reported in Figure 2 – figure supplement 2C, Figure 4E, F and Figure 5C.
- In Validation of LM-based bouton detection methodology with EM subsection of Results we report the sample size and Pearson’s r.
- In Effects of imaging conditions on bouton analysis subsection of Results and Figure 5 we report sample sizes, statistical tests, and p-values.
- In Statistical framework for the analysis of structural plasticity of boutons subsection of Results we perform one sample KS test and report p-values.

(For large datasets, or papers with a very large number of statistical tests, you may upload a single table file with tests, Ns, etc., with reference to sections in the manuscript.)

**Group allocation**

- Indicate how samples were allocated into experimental groups (in the case of clinical studies, please specify allocation to treatment method); if randomization was used, please also state if restricted randomization was applied
- Indicate if masking was used during group allocation, data collection and/or data analysis

Please outline where this information can be found within the submission (e.g., sections or figure legends), or explain why this information doesn’t apply to your submission:

Group allocation is irrelevant for this Tools and Resources paper.

**Additional data files (“source data”)**

- We encourage you to upload relevant additional data files, such as numerical data that are represented as a graph in a figure, or as a summary table
- Where provided, these should be in the most useful format, and they can be uploaded as “Source data” files linked to a main figure or table
- Include model definition files including the full list of parameters used
- Include code used for data analysis (e.g., R, MatLab)
- Avoid stating that data files are “available upon request”

Please indicate the figures or tables for which source data files have been provided:

Data used in the CLEM experiment are available in the Dryad Digital Repository (doi:10.5061/dryad.3q50t). This dataset includes a 2PLSM image stack in TIFF format, optimized traces of four axon segments in SWC format, and 3D EM reconstructions of these axons in Blender format.

Source code and user manual of BoutonAnalyzer software are available on GitHub repository (<https://github.com/neurogeometry/BoutonAnalyzer>).

BoutonAnalyzer demos, user manual, and sample data are available at <http://www.northeastern.edu/neurogeometry/resources/bouton-analyzer> .

Tools for measurement of axon cross-section areas and bouton volumes in EM reconstructions have been integrated with the NeuroMorph toolset, available at <https://github.com/ajorstad/NeuroMorph>.
